# Impact of the Macmillan specialist Care at Home service: a mixed methods evaluation across six sites

**DOI:** 10.1186/s12904-018-0281-9

**Published:** 2018-02-23

**Authors:** Bridget Johnston, Anne Patterson, Lydia Bird, Eleanor Wilson, Kathryn Almack, Gillian Mathews, Jane Seymour

**Affiliations:** 1 Florence Nightingale Foundation Professor of Clinical Nursing Practice Research, School of Medicine, Dentistry and Nursing, University of Glasgow, 57-61 Oakfield Avenue, Room 61/504, Glasgow, G12 8LL UK; 2 School of Sociology and Social Policy, University Park, University of Nottingham, Nottingham, NG7 2RD UK; 3 Present address: Division of Primary Care, University of Nottingham, Queens Medical Centre, Derby Road, Nottingham, NG7 2HA UK; 4 School of Health Sciences, University of Nottingham, Queens Medical Centre, Derby Road, Nottingham, NG7 2HA UK; 5 School of Health and Social Work, University of Hertfordshire, Hatfield, Hertfordshire, AL10 9AB UK; 6 School of Medicine, Dentistry and Nursing, University of Glasgow, 57-61 Oakfield Avenue, Glasgow, G12 8LL UK; 7 School of Nursing and Midwifery, The University of Sheffield, Barber House Annex, 3a Clarkehouse Road, Sheffield, S10 2LA UK

**Keywords:** (Macmillan) specialist palliative care service, End-of-life care, Patient choice, Complex intervention, Mixed methods evaluation

## Abstract

**Background:**

The Midhurst Macmillan Specialist Palliative Care at Home Service was founded in 2006 to improve community-based palliative care provision. Principal components include; early referral; home-based clinical interventions; close partnership working; and flexible teamwork. Following a successful introduction, the model was implemented in six further sites across England. This article reports a mixed methods evaluation of the implementation across these ‘Innovation Centres’. The evaluation aimed to assess the process and impact on staff, patients and carers of providing Macmillan Specialist Care at Home services across the six sites.

**Methods:**

The study was set within a Realist Evaluation framework and used a longitudinal, mixed methods research design. Data collection over 15 months (2014–2016) included: Quantitative outcome measures - Palliative Performance Scale [PPS] and Palliative Prognostic Index [PPI] (*n* = 2711); Integrated Palliative Outcome Scales [IPOS] (*n* = 1157); Carers Support Needs Assessment Tool [CSNAT] (*n* = 241); Views of Informal Carers –Evaluation of Services [VOICES-SF] (*n* = 102); a custom-designed Service Data Tool [SDT] that gathered prospective data from each site (*n* = 88). Qualitative data methods included: focus groups with project team and staff (*n* = 32 groups with *n* = 190 participants), and, volunteers (*n* = 6 groups with n = 32 participants). Quantitative data were analysed using SPPS Vs. 21 and qualitative data was examined via thematic analysis.

**Results:**

Comparison of findings across the six sites revealed the impact of their unique configurations on outcomes, compounded by variations in stage and mode of implementation. PPS, PPI and IPOS data revealed disparity in early referral criteria, complicated by contrasting interpretations of palliative care. The qualitative analysis, CSNAT and VOICES-SF data confirmed the value of the Macmillan model of care but uptake of specialist home-based clinical interventions was limited. The Macmillan brand engendered patient and carer confidence, bringing added value to existing services. Significant findings included better co-ordination of palliative care through project management and a single referral point and multi-disciplinary teamwork including leadership from consultants in palliative medicine, the role of health care assistants in rapid referral, and volunteer support.

**Conclusions:**

Macmillan Specialist Care at Home increases patient choice about place of death and enhances the quality of end of life experience. Clarification of key components is advocated to aid consistency of implementation across different sites and support future evaluative work.

**Electronic supplementary material:**

The online version of this article (10.1186/s12904-018-0281-9) contains supplementary material, which is available to authorized users.

## Background

Finding new models of palliative and end-of-life care to meet the needs of an increasingly co-morbid and ageing patient population is an international challenge [1]. In England and Wales, most deaths occur in hospital (around 53%) despite the preference of most people to be cared for and to die either at home (63%) or in a hospice (29%) [2]. High numbers of patients with conditions that give rise to palliative care needs are admitted to hospital as emergencies [3], often with problems and symptoms that are potentially amenable to management in the community. Care of the dying at home is associated with a range of clinical and social complexities [4–6]. A national survey of bereaved people in England highlighted the importance of family carer support with 50% of responders recalling a need for more help during the person’s illness [7]. Systematic reviews show that home based palliative care results in higher satisfaction among patients and caregivers, reduces hospital usage towards the end of life and, compared to death in hospital, is associated with less intense grief amongst family carers and a greater sense of peace [6] in the last week of life for patients.

The likelihood of receiving good quality palliative care at home is subject to variation by condition, by age and by region and it is estimated that a minimum of 63% of all deaths need palliative care [8], a service that is still mostly delivered to people with advanced cancer [9]. NHS expenditure on specialist palliative care services varies significantly across different regions, with evidence suggesting an excess of a 30 fold variation in expenditure. [10] There is no national definition of what counts as a ‘specialist palliative care service’ and there is a lack of standardised referral practices [11–13].

In this paper, we present key aspects of a mixed methods evaluation in six sites across England (hereafter ‘Innovation Centres’) of a new model of community based palliative care: ‘Macmillan Specialist Care at Home’. The Programme aims to establish sustainable and affordable palliative care in the community, following four broad principles:

- Establishing patterns of early referral,
- Delivering clinical interventions at home where possible,
- Ensuring coordinated care through collaboration between service providers,
- Encouraging flexible teamwork between specialists, generalists and volunteers [14].

This evaluation aimed to assess the process and impact on staff, patients and carers of providing Macmillan Specialist Care at Home services across the six Innovation Centres over a 15-month period. A summary report can be accessed from the Macmillan website [15] and full report on request.

### Macmillan specialist care at home

The multi-site implementation of Macmillan Specialist Care at Home is an extension of the Community Specialist Palliative Care Service launched in Midhurst in 2006, the origins of which are rooted in the Swedish Motala Home Care model [16]. The aim is to maximise holistic delivery of palliative care services in the home/community setting. Key Key features of Macmillan Specialist Care at Home [15] are:

1. Early referral - People are referred to the multidisciplinary team, often while still having active treatment. This allows enough time to build strong relationships, plan ahead and provide practical and emotional support when needed.
2. Home-based clinical interventions - A broad range of interventions (including blood/blood product transfusions, IV antibiotics or bisphosphonates, ultrasound, intrathecal analgesia) can be provided at home or in a community setting. This can be less stressful for patients and carers, and saves time and energy for other activities.
3. Close and proactive collaboration with primary care and other service providers - Better coordination between services and service providers is key to a better experience of care. Collaboration and joint working is central to this approach.
4. Flexible teamwork between specialists, generalists and trained volunteers - The flexibility of roles undertaken by team members is reported as key by patients, carers and staff. Research shows that caregivers value accessibility and support, and patients emphasize the psychosocial aspects of services.

The programme was piloted and evaluated in Midhurst, a rural area in the south of England [14]. The evaluation revealed that a higher percentage of patients (71%) died at home, a significant increase compared to elsewhere. Patients and carers reported good quality of care and qualitative interviews showed strong, collaborative team working with other services. Earlier referral into the service prevented around 20% of total care costs in the last year of life through reduced the use of hospital inpatient services and Accident and Emergency department attendances [14].

Macmillan Cancer Support were keen to build on this success and to establish whether key components from the pilot could be effectively transferred, and outcomes replicated, on a national scale and across a diverse range of communities. To test this hypothesis six Innovation Centres received funding to support service development in line with the key features of Macmillan Specialist Care at Home.

## Methods

### Study design

The study drew on Realist Evaluation principles following Pawson and Tilley’s thesis that material and social worlds are real and exert influence on outcomes [17]. The approach is grounded in Critical Realism which recognises the multi Layered nature of the social world and its effect on human behaviour [18]. Critical Realism pursues the middle ground between relativism and positivism, positing that the natural and social sciences are logically compatible [19]. The realist view draws on both qualitative and quantitative techniques to engage with the interactive processes that connect both material and social realities [20, 21]. Evaluating the effectiveness of complex interventions in a real world context presents a major challenge because of the multifaceted, interconnected elements involved [22]. For research purposes, this is often achieved through a mixed methods design that draws on both qualitative and quantitative research techniques. Realism is viewed as a general research strategy rather than a strict technical procedure [23]. It assumes a theoretical perspective that not only tests hypotheses about whether or not a programme works, but also seeks to reveal the contributory mechanisms that bring about change [24]. Working from this premise the question is: “What works for whom, and under what circumstances?” placing focus on a mechanism-context-outcomes configuration [17].

### Setting and participants

The six Innovation Centres represented a diverse cross-section of urban, rural and island populations, with a range of ethnic minority groups. The sites selected were weighted towards areas with higher levels of deprivation and chronic disease incidence and prevalence, lower than national averages for life expectancy and rates of home death. Participants included staff, volunteers, patients and carers. Table 1 identifies key similarities and differences of the sites.

**Table 1:** Innovation Centres and identified focus areas

Site A
Develop volunteer provision to support home visits to patients
Streamline service delivery across the two areas
Establish a local primary care learning network to widen knowledge about palliative and end of life care
Develop protocols and deliver clinical interventions at home
Site B
Create a single point for access for referral
Fund additional personnel, in particular two part-time consultants in palliative medicine
Work to integrate community teams and create a central ‘hub’
Develop systems for early referral
Develop protocols and deliver clinical interventions at home
Site C
Fund a speciality doctor to set up another palliative care clinic
Work with local care homes to provide education and support
Create an end of life education programme
Develop volunteer provision to support home visits to patients
Develop protocols and deliver clinical interventions at home
Site D
Fund additional personnel, including community support workers and an occupational therapist
Work to integrate community teams
Develop systems for early referral
Increase rapid response and 24/7 access
Develop protocols and deliver clinical interventions at home
Site E
Fund additional personnel, including a staff grade doctor, advanced nurse practitioner and two part-time health care assistants (HCAs)
Develop systems for early referral
Develop as rapid response team of HCAs
Develop volunteer provision to support home visits to patients
Develop protocols and deliver clinical interventions at home/hospice
Site F
Fund additional personnel, in particular a nurse consultant
Create a single point for access for referral
Develop volunteer provision to support home visits to patients
Work to integrate community teams

### Data collection

Additional file 1 highlights the data collection methods used in the study. Data were collected at three time points: Baseline- as the project was being set up; Interim; and, Final, evenly spaced over a 15 month period.

### Qualitative data

#### Focus groups

Focus groups (*n* = 32) were undertaken at three time points: baseline, interim and final. These were conducted with staff, who included those managing the implementation of the projects, local stakeholders (e.g. commissioners) and front line clinical nursing and medical staff (*n* = 190). Six additional focus groups were conducted with volunteers (*n* = 32) at interim and final time points.

The focus groups allowed for a discursive format in which participants created a consensus narrative about the process and outcomes of the implementation of Macmillan Specialist Care at Home including wider impacts on palliative and end of life care. This method of data collection enabled participation of those who might not have been comfortable being interviewed by themselves or who may have felt they had little to contribute [25].

Variations in focus group design were based on the different participant groups, numbers taking part, and, the nature of their knowledge base [26]. Staff were asked about service developments, working patterns and methods whilst volunteers were asked about their perceptions and experiences of their roles. Group sizes varied from 2 to 10 participants and at two centres in the final stage, staff were unavailable to participate and individual interviews were conducted with the project managers.

#### Individual interviews

Individual interviews averaging 50 min each were conducted with staff (*n* = 18), volunteers (*n* = 7), volunteer managers (*n* = 7), patients (*n* = 9), and, carers (*n* = 8) (total = 49). These were conducted in the interim project phase between June 2015 and September 2015 (the main outcomes from this study are in the process of publication). Pictor [27], a visual technique, was used to facilitate dialogue, by inviting the participant to create a simple visual representation of experiences of care. The ‘Pictor’ process begins by inviting the participant to recall a palliative care case that they can elicit clearly (or in patient and carer interviews, their own case). Participants are given a large blank sheet of paper (A1 size) and arrow-shaped ‘Post-It’ notes. They are then asked to write on the arrows the initials, role title or a pseudonym for every person they can remember who had some involvement in the case including themselves and the patient. There are no fixed rules as to how the participant places the arrows, but they are encouraged to use aspects such as the direction of the arrows and proximity to other arrows to indicate features of relationships in the case. The task is completed alone to help reduce researcher influence. Once the chart is completed, the interviewer uses it as a focal point for case discussion.

### Quantitative data

The quantitative data investigated patient and carer outcomes. Prospective data was also obtained to develop standardised evaluation measures for the individual sites:

#### Service data tool [SDT]

The SDT was developed by the evaluation team in consultation with the Innovation Centres and Macmillan Cancer Support at commencement of the evaluation in April 2014. It was implemented monthly from November 2014 for a period of 15 months with data (*n* = 88) fed back to the evaluation team via Survey Monkey©. It collated service level data on: 1) referrals; 2) place of death; 3) Staff activity logs; 4) number and type of clinical interventions; 5) location of the intervention delivery.

#### Palliative performance scale [PPS] and palliative prognostic index [PPI]

These scores were used to give an indication of symptom burden, physical function and expected survival time upon referral to the service. Palliative Performance Scale (PPS) is a modification of the Karnofsky Performance Scale (KPS), developed in 1996 and validated as a reliable tool [28]. It is designed specifically for measurement of physical status in palliative care. Using the PPS, only about 10% of patients with a score of 50% or less would be expected to survive more than 6 months. A score was recorded for each patient who was assessed on referral to Macmillan Specialist Care at Home, alongside the PPI.

Palliative Prognostic Index (PPI) [29, 30], also a validated tool, was used alongside the PPS assessing oral intake, oedema, dyspnoea at rest and delirium. If the PPI is greater than 6.0, survival is estimated to be less than 3 weeks. The aim was for this measure to be completed by the nurse on first visit to the patient on referral to Macmillan Specialist Care at Home alongside the PPS. These outcome measures were returned by the sites on a monthly basis.

#### Integrated palliative outcome scales [IPOS]

The IPOS is a validated instrument that combines the best elements of the Palliative Care Outcome Scale (POS), the additional symptom module and the African version of the outcome scale to measure physical symptoms, psychological, emotional, spiritual, information and support needs [31]. The IPOS tool was taken to the patient’s home by a nurse from Macmillan Specialist Care at Home and completed by patients on each visit (maximum once a week) to identify symptom burden.

#### Carers support needs assessment tool [CSNAT]

The CSNAT tool was developed and validated by the Universities of Manchester and Cambridge to assess carers’ needs in home based end of life care contexts [32, 33]. While the tool is designed to capture change over time in carers’ needs, in this evaluation, it was not feasible to do so and the questionnaire was issued to current carers on just one occasion in the final period of data collection to achieve a cross sectional picture of their needs at that time point.

#### Views of informal Carers – Evaluation of services [VOICES-SF]

The VOICES-SF (Views of Informal Carers – Evaluation of Services: Short Form) [34, 35] is a self-report measure designed to capture experiences of end of life care that can detect differences between service providers, care settings, and place of death. The questionnaire was used to gather the views of bereaved carers (3 months post-bereavement) of patients who had received services from the Innovation Centres. Specific objectives were: 1) to discover how many carers knew of a preferred place of death for the person they were caring for; and, 2) what percentage of patients, who had expressed a preference for where they wanted to die, achieved the preference expressed?

### Qualitative data analysis

The qualitative data was examined using thematic analysis. The qualitative data sets consisted of focus groups, Pictor [27] interviews and qualitative responses within the CSNAT and VOICES-SF questionnaires. All interviews and focus groups were digitally recorded and transcribed verbatim with all patient identifying data removed. The qualitative data from the CSNAT and VOICES-SF questionnaires was also transcribed. During all site visits field notes were used to record observations and make additional notes about the interviews and focus groups. These data were used to provide a contextual backdrop for the study and to inform further areas of exploration at subsequent data collection visits.

Interviews were then analysed using an inductive thematic analysis, [36] which involved familiarisation with the data, development of a coding framework and theme development. A preliminary coding framework was developed through discussion at evaluation team meetings and on the basis of the initial evaluation aims. Several members of the evaluation team coded data sources separately. Codes were then compared and discussed in order to group into themes and then further distilled into categories. This was an iterative process, refined as data collection proceeded and ultimately a final coding framework was applied to all data. Analysis was multifaceted, working across and within data sources from all six Innovation Centre sites to capture, in thematic format, the views and experiences of all participants and stakeholders involved in the projects. This enabled identification of issues of relevance to the different groups. Elements of the data were presented to the Innovation Centre teams throughout the evaluation period during regular ‘Community of Practice’ events. These events allowed the evaluation team to check the validity of the ongoing analysis and emergent themes. Our analysis was informed and underpinned by a Critical Realist approach [37, 38] to make sense of the different ways in which participants actively constructed and accorded meaning to the ‘reality’ of their experience. The purpose of the qualitative interviews was to unpick the deeper layers of experience to help identify causal mechanisms and their effects within the social context of home-based palliative care provision. In particular, the analysis explored the challenges of implementing the service and how some of its key features - early referral, clinical interventions at home, the avoidance of unscheduled hospital admissions, and, enabling death at home, occurred in practice.

### Quantitative data analysis

All data was cleaned prior to examination. The SDT information was exported from Survey Monkey© into Microsoft Excel 2010© and scrutinised for: 1) The number of referrals made to the services; 2) The nature and volume of activity that was carried out at each site; 3) The total amount of time required from different health professionals and volunteers to provide different types of activity; 4) The number of patients who received specific interventions each month; 5) The number of people who died at each site; 6) The number of people who died at home. Means were calculated for the amount of time required from different health professionals and volunteers to provide one instance of each type of activity and the number of times an activity was carried out per patient.

Analyses of PPS and PPI, CSNAT, VOICES-SF and IPOS questionnaires was performed using SPSS software version 21. Response rates were calculated and the characteristics of respondents, described. Calculations were carried out to establish: frequencies and percentages for all categorical variables; mean and standard deviation for normally distributed data; median and inter-quartile range for skewed data. The normality of continuous measures was checked using histograms to provide a good empirical description of the variables in accord with the procedures of statistical analysis [39]. The mean PPI/PPS score and indicative patient survival time at referral were calculated monthly for each site and overall**.** IPOS data was used to calculate the frequency and percentage of responses (at referral) to each item of the scale.

In order to provide insight into factors that may affect whether patients receive their final care at home (a key objective of the service), a univariate analysis was used to test for association between: place of death, and patient/carer characteristics, gender, age, and dying in their preferred place of death to ascertain whether factors such as gender, age and relationship exerted impact on this; length of time Macmillan Specialist Care at Home had been established; PPI/PPS scores at referral; and, IPOS scores and length of time patients received care from Macmillan Specialist Care at Home. T-tests assessed normal continuous data and a significance level of 0.05 was used to test the significance of association. A univariate regression was performed to estimate the effect size of association between length of time receiving care from Macmillan Specialist Care at Home and IPOS score, with 95% confidence intervals. Data were collected at irregular (non-systematic time points) and unbalanced (different time intervals) for each patient. This meant the repeated measures aspect of the data could not be fully exploited; a linear regression was, therefore, used.

### Ethical considerations

Ethics approval for the study was granted by the Faculty of Medicine and Health Sciences Research Ethics Committee, University of Nottingham. Participants provided written consent following a verbal explanation and receiving written information on the study.

To ensure confidentiality, qualitative interviews and field notes were anonymised. Where required, permissions to use the assessment tools were obtained. Questionnaires were distributed to patients and carers by Innovation Centre staff. The VOICES-SF tools were posted directly to the bereaved carers’ homes. All measures were accompanied by an information sheet and covering letter, headed by the University of Nottingham and the relevant NHS Trust. Prepaid envelopes, with a return address to the evaluation team at the university, were included. Other measures were collated by the Innovation Centre staff. All identifying data were removed from the completed forms and replaced with pseudonyms. The Innovation Centre code and the date were added before the completed forms were sent to the evaluation team. The knowledge and expertise of the research team was applied to ensure that data collection methods and the conduction of data collection were sensitively tailored to maximise benefit and minimise risk to participants. An independent advisory board met at regular intervals throughout the study, to monitor progress and respond to any study issues.

## Results

Findings are drawn from the analysis of data gathered from staff, volunteers, patients and carers from all six Macmillan Specialist Care at Home Innovation Centres and relate to patients referred over a 15 month period between 01/11/14 and the 31/01/16.

Additional file 2 provides details of the qualitative and quantitative data gathered from each Innovation Centre and the response rate for completion of the tools. Not all Innovation Centres were able to provide all forms of data. In part, this was the result of varied priorities.

### Quantitative data

#### Service data tool [SDT]

The demographic and clinical details of patients who were treated by Macmillan Specialist Care at Home are shown in Additional file 3. The mean age of patients across all sites was 75.7 years and just over 50% (*n* = 1655) of the patients were men. Approximately a third [*n* = 3229, (32.5%)] had a non-cancer primary diagnosis.

#### Referral patterns

In total *n* = 3286 patients were referred to the six different Macmillan Specialist Care at Home centres during the evaluation period (see Additional file 4 for further details about referrals to each site). The data indicates that whilst the vast majority of patients [*n* = 3041, (94.2%)] referred to Macmillan Specialist Care at Home went on to receive care from that service, *n* = 145 individuals (4.4%) died after being referred to Macmillan Specialist Care at Home before any care could be delivered. A wide variation in numbers of patients referred was noted across the Innovation Centres (Additional file 4) with Site B service receiving the highest number (*n* = 1998) during the evaluation period whilst Site A received only 15. A small number of referrals were not accepted due to patients not having palliative care needs. In part, the variation reflects the differential focus that each Innovation Centre placed on specific aspects of Macmillan Specialist Care at Home (see Box 2) and the stage of project implementation.

#### Specialist and other supportive interventions

Across the six Macmillan Specialist Care at Home services a range of clinical procedures were undertaken. Eighty per cent were delivered in the home environment and required substantial medical (*n* = 97 h), nursing (*n* = 223 h) and health care assistant (*n* = 116 h) input. The predominant focus was on phlebotomy with a limited number of the more complex interventions such as paracentesis and cannulation. Support was also given via a substantive range of services comprising: volunteer visits (*n* = 3804 h); day services (*n* = 1227 h); telephone (*n* = 2623 h); and, routine and rapid response home support (*n* = 7089 h). Volunteers undertook a range of activities including befriending, housework, shopping and transport whilst day services offered medical and social support. Telephone calls were mainly concerned with queries around care packages and symptom management. Routine and rapid response support in the home enabled patients to stay in their preferred place of care and avoid unplanned hospital admissions. Although, this involved medical and nursing interventions to provide, for example, emergency prescribing, the role of health assistants in rapid response was significant. Their input focused on alleviating patient and carer anxieties and relaying information to other, more senior, members of the team. Findings show that whilst specialist interventions were the anticipated focus of the new services, considerably more staff time was spent delivering conventional support to patients.

#### Palliative performance scale [PPS] and palliative prognostic index [PPI]

Data were received from participants [*n* = 2711, (response rate 88.8%)] across all Innovation Centres but PPI data from Site A was not analysed due to a high percentage (87.6%) of missing data. Collectively, the PPI/PPS data gives insight into the timing of referrals. The PPI scores, generated by combining PPS scores with additional clinical information was used to provide an indication of expected survival time. The PPI scores suggest that referral into Macmillan Specialist Care at Home (see Additional file 5) is taking place either when patients have an indicative life expectancy of more than 6 weeks [*n* = 1278, (51%)] or less than 3 weeks [*n* = 939, (37.5%)] with comparatively few referrals occurring between 3 and 6 weeks [*n* = 290, (11.6%)].

#### Integrated palliative outcome scales [IPOS]

IPOS data were returned for a total of 1157 patients across five Innovation Centres. Additional file 5 shows the symptom burden reported by patients when first completing the IPOS questionnaire. Weakness and poor mobility were the most reported symptoms. Such challenges to patients’ mobility may support the drive towards providing specialist care and interventions within the home environment. Although these data suggest that most people are referred before their symptoms are severe, it is notable that some people reported ‘severe’ or ‘overwhelming’ symptoms. The majority of this sub-group of patients reported that they were affected, to some extent, by at least half of the key IPOS symptoms illustrated in Additional file 6 (below) highlighting a need for further work to ensure that people are admitted to the service at a time when appropriate planning can be put in place for their support. The data from Site E appears to indicate that a widening of referral criteria to capture those with palliative care needs during their illness promoted earlier referral to Macmillan Specialist Care at Home when patients were still experiencing a relatively low disease burden.

Analysis of IPOS data over time showed a small, non-statistically, significant decrease in symptom burden (*p* = 0.3120). The mean difference between first and last IPOS scores was (− 0.05 to 0.16). Assessing the relationship between the mean IPOS score with time receiving Macmillan Specialist Care at Home services (continuous) showed a small statistically insignificant correlation of − 0.09 (*p* = 0.1223). Further, univariate regression showed a statistically insignificant reduction in mean IPOS score of − 0.0005 (95% CI: -0.0012 to 0.0001) per day increase in the service. While our analysis of IPOS data did not indicate any statistically significant findings it is encouraging that symptom burden was maintained or even reduced as people remained in the service and, therefore, by definition as they neared death.

#### Place of death

Over the project period, *n* = 3054 patients were referred into Macmillan Specialist Care at Home. Of these, *n* = 2127 died, *n* = 1085 deaths occurred at home (51%), *n* = 316 in a hospice (14.8%), *n* = 627 (29.5%) in hospital, *n* = 74 (3.5%) in other locations and place of death is recorded as unknown for *n* = 77 (3.6%) patients. Place of death broken down by site is shown in Additional file 7. Despite having a higher proportion of ‘late’ referrals Site F achieved the highest number of home deaths. The integration of their HCAs and Hospice at Home teams appears to have impacted positively on this outcome. Site D also achieved a high proportion of home deaths. At both sites D and E where services were hospice based, SDT data revealed a change in the pattern of in-patient hospice use whereby more care took place at home even when preferred place of death was the hospice.

#### Carers support needs assessment tool [CSNAT]

The CSNAT questionnaire, which explores the impact of caring on current carers, was completed by *n* = 241 carers across five of the six Innovation Centre sites. The majority of questionnaires were returned by carers of people accessing Macmillan Specialist Care at Home at Site B [*n* = 180/241 (75%)] which recorded the highest number of referrals (see Additional file 4) and therefore, had more carers to whom the questionnaire could be sent. Findings are summarised in Additional file 8 below. The data shows that, in general, carers felt their needs were well met by the service and did not require a lot in terms of further input. A few areas of outstanding need were, however, identified. Carer responses to the question: ‘Do you need more support with knowing what to expect in the future when caring for your relative?’ indicated that more assistance in this area would be welcomed with 58.9% of respondents (*n* = 237) requesting either ‘a little more’ (35.9%), ‘quite a bit more’ (14.8%), or ‘very much more’ (8%) support. Other areas where additional help for carers was indicated comprised: how to deal with their feelings; knowing who to contact; understanding and talking about illness with their relative. In addition, approximately one quarter of the sample expressed a desire for more input on personal health, financial and legal issues, and practical help in the home. In contrast, very few people related a need for spiritual care.

#### Views of informal Carers – Evaluation of services [VOICES-SF]

Demographic details amassed from the *n* = 102 carers responding to the VOICES-SF questionnaire showed that the large majority of respondents (70%) reported that they were the spouse of the patient, and 20% described their relationship as son/daughter of the patient.

The VOICES-SF questionnaire asked bereaved carers to rate the care that they had received from Macmillan Specialist Care at Home (Additional file 9). A small number [*n* = 11, (10%)] did not recognise that they had received care from this service, highlighting the difficulty that patients may experience in differentiating care from different providers. For those who did recognise receiving input from Macmillan Specialist Care at Home, when asked about the overall quality of care, the majority answered ‘Exceptional’ or ‘Excellent’ [*n* = 69, (68%)]:

Only 4% (*n* = 4) felt that the care had been ‘fair’ or ‘poor’. The high satisfaction level was reflected in findings that showed that [*n* = 76, (75%)] of carers felt they had been involved in decisions about treatment as much ‘as they would have liked’, with [*n* = 66, (65%)] reporting that they received as ‘much help and support as needed.’

Only 5.9% (*n* = 6) of respondents felt they had not received enough help and support at the time of the patients’ death. The preferred place of death of the person they were caring for was known by 61.8% (*n* = 63) carers. Where a preference for place of death was known, [*n* = 81, (79.4%)] of patients had died in that preferred place. Univariate analysis of patient and service characteristics did not indicate that any variables were significantly associated with dying in a particular location. Furthermore, a high percentage [88.3%, (*n* = 90)] of respondents felt that the person they cared for had died in their preferred place of death.

### Qualitative data

We report findings from the qualitative data according to the insights they provide about the implementation of the four broad key principles underpinning Macmillan Specialist Care at Home: establishing patterns of early referral, delivering clinical interventions at home; ensuring collaboration between service providers, and encouraging flexible teamwork.

#### Establishing patterns of early referral

The qualitative data confirmed the imprecise nature of ‘early referral’. A lack of standardised criteria across the sites made it challenging for the evaluation team to interpret how each Innovation Centre was progressing in terms of achieving ‘early’ referrals. The quote below from a staff member at Site B, however, provides an illustrative and comprehensive summary of what an early referral could look like. It does so from a patient-focussed perspective and identifies that a key measure of success is getting support in place before crises occur, and, provides something of a benchmark to which services might aspire:

*So many of the referrals that we get are sort of virtual crisis referrals, other things have fallen down. So [an early referral would allow time to do] baseline assessment because you can properly see how things will change. And you can get a measure of the person and their normal support structure, their normal coping skills, without a number of external influences that makes something a crisis. So that for me would be an early referral, just evidence that someone was thinking ahead. (Site B, staff focus group, baseline).*

A small number of bereaved respondents provided qualitative responses in the VOICES-SF survey questionnaire, suggesting that in some cases patients were only referred in the last days of life:

*I cannot complete much of this survey as we were only introduced to your organisation 4 days before he died – but we did appreciate your care during that time. Earlier support would have been helpful. (Site F, VOICES*-SF*, Carer 7)*

*I only had help with my husband at night for two nights before he died, as I was tired out and needed to get some sleep. I could have done with more help in that respect, and also when he needed a shower. I could have done with some help. (Site D, VOICES-SF, Carer 12)*

Staff identified situations where wider referral systems and processes had the propensity to address the challenges of capturing early referrals. At the outset it was noted that, where there were various referral points into services, there was also the potential for people to ‘fall through gaps’ and/or be subject to differing standards of ‘gatekeepers’ with different criteria for referring patients to services:

*I feel that there are huge gaps for patients, once they are not in the system for curative care or curative treatment, they fall in a huge gap and the GP is not always really on the ball. So I feel that I am constantly seeing patients who I think, ‘I can’t believe this’, why not earlier referral, why not, why are we just constantly mopping and taps are running all over the place and everyone is really just mopping hard. (Site C, Staff focus group, baseline)*

Staff recognised a need to broaden traditional perceptions of palliative care as a service that is offered to critically ill patients approaching death, to include those living for longer periods with chronic, debilitating illness. This facet was accorded importance, not only to establish the service but also to increase the number of ‘early’ or ‘timely’ referrals in line with Macmillan’s aims for specialist palliative care provision. Participants considered the need to raise patient and professional awareness of what the service could offer:

*I think that it’s all about education of GPs and district nurses in that, still the perception is about hospices and specialist palliative care for patients who are dying in their last couple of weeks of life. And that’s just a small part of what we do, and it’s out of date …We’re having so many more patients with long-term conditions, neurological conditions, and specialist palliative care is complex symptom control management, and that’s where our expertise is. (Site A, Project team focus group, baseline)*

Where a single point of access was introduced (Sites B, C and D), it was reported to improve responsiveness and direct patients to the most appropriate services at the most appropriate time. Patient preference, however, could prevent staff intervening in what was perceived to be a timely manner:

*Not everybody wants (us), I spoke to another 92 year old man today who wants to stay at home* (to die)*, but he’s not ready to talk to us yet. That will get to crisis, we will get a phone call, and it’s that idea that people think that we haven’t bothered - whereas we have, but at that time it wasn’t what he wanted. (Site B, Staff focus group, baseline)*

Further, the desire to increase early referral into palliative care and end of life services was juxtaposed against a realist view of what and how much could be offered if there was an exponential increase in demand.

#### Delivering clinical interventions at home

Although a key purpose of Macmillan Specialist Care at Home is to test the introduction of specialist interventions into the community, due to local factors, these were not initiated at every site. The data showed that where they were carried out, these constituted a small part of the overall service. At the baseline interview, site D staff anticipated being able to administer blood transfusions and bisphosphonates in patients’ homes and it transpired that they did conduct the highest number of this type of procedure including paracentesis. The project team, however, reported a limited demand, overall, for these complex interventions:

*What we haven’t done much of …the blood transfusions and the bisphosphonates and things like that. I still think it will be useful, don’t get me wrong, and I think it’s something to have in our bag to do. And the time we did the bisphosphonates the consultant went out with [the nurse] and they did it themselves. And that was really useful, because this chap couldn’t get up to the hospital or [the hospice]. So that was really useful. And we do subcut fluids and that, that’s just as and when really. I want to carry on with that, and I want to actually have it as part of the team, although I don’t see it as a huge necessity. (Project team focus group, final)*

Complex interventions take significant time and input to embed within service delivery suggesting the need to weigh up their provision against palliative care team resources. Regardless of this service component, participant accounts reveal that a substantial range of services were made available to patients and their families. In particular, the extent of routine and rapid response home support enabled patients to stay in their preferred place of care and avoid unplanned hospital admissions:

*We’ve had quite a few [patients] lately that the team have come out and helped us with, and we’ve managed to keep them at the home rather than sending them into hospital. (Site C, Staff focus group, final)*

#### Ensuring collaboration between service providers

The Innovation Centres saw part of their role as working across services to pull the threads of end of life care together to offer a coordinated service. A patient summed up the service-user perspective:

*I had the feeling that somewhere behind the scenes it was all superbly coordinated... it just gave me the impression, and indeed the reassurance, that people who were looking after my interests were working as a team...it all seems to have been put together so that at the end of the day we’ve got a jigsaw puzzle with five hundred pieces in place. (Site B, Pictor, Patient)*

Staff and volunteers perceived that local project ‘champions’ - often a dedicated project manager- helped to implement the project and to coordinate and manage activities. These motivated individuals, who maintained an overarching perspective on proceedings, offered a central point of contact for the team and formed critical links with partner organisations. Strong leadership along with education and training were highlighted as fundamental in preparing staff to work together to implement Macmillan Specialist Care at Home. Many staff and volunteers subsequently reported the development of new and improved relationships with other local professionals, aspects that were perceived to impact on their job satisfaction and bolstered enthusiasm to ensure the provision of quality care.

#### Encouraging flexible teamwork

For some Innovation Centres a key goal was to integrate existing palliative care service provisions by bringing together hospice, hospital and community services. Where several teams were providing similar services, this proved challenging and, particularly in locations where end of life care provisions were already well developed, a level of concern was expressed about the potential ‘dilution’ of established services. Furthermore, at the start of the implementation process, some of the existing staff expressed apprehensions about the impacts of the new service on their role:

*I think from a personal point of view I think it’s difficult to see how things, how my role could change really, or if I want it to change. (Site B, staff focus group, baseline)*

Dealing with staff trepidation required sensitive and careful management, especially where there was a degree of historical antagonism. This situation was more prevalent where there was some overlap between the role of the Macmillan team and current community staff. Key enabling factors for progressing integration were to sensitively pace the process and to bring together the relevant staff at an early stage. In this regard, joint education and training were pivotal, supporting positive relationship building and establishing common ground:

*We all contribute and we all deliver to the same programme…we’ve got champions in every area, and they’ve all got the same end of life competencies, so if they work in different settings and are mentored by different disciplines, they will know the common goal of what those competencies are, and for the patient moving through, the staff have had the same education. (Site B, staff focus group, final)*

The inclusion of consultants in palliative medicine was attributed high value as they could expedite clinical decision-making and offer effective patient care at the point of need. These senior practitioners provided a source of common support which engendered staff confidence and exerted a positive influence on community partnerships:

*...the biggest change was the consultants coming. I think that’s had a huge impact on the team. Personally I feel much more supported...It is having that ready access to them, and them just being so amenable. (Site B, Pictor, Therapist)*

The value of health care assistants (HCAs)^1^was also frequently highlighted by staff and project team members. Sites commonly reported that HCAs had the ability to respond quickly to patient need, often maintaining a situation until a specialist assessment could be made. The HCAs, thus, appear to be instrumental in ‘joining up’ provision. They were often seen as filling some of the gaps in service provision, particularly out of hours, and providing the rapid response necessary whilst other services were being put in place. Overall, sites valued HCAs for providing:

- Continuity for patients and ‘joined up’ care
- Feedback to other team-members in their capacity as ‘eyes and ears’ of the team in patients’ homes
- Rapid response and fending off of potential crises by ‘holding’ situations whilst a solution could be found:

*Because they’re on the ground so to speak, they’re there, they’re with the patients, they’re spending their time, their understanding is there. …makes it a lot easier to pass on that information to the right person whether it’s social work or CNS (clinical nurse specialist) and then get the support that the patient needs that way. (Staff focus group, final).*

It was noted, however, that HCAs need to be managed and supported by experienced qualified/ registered staff. In general, though, the components of skill mix and flexible team working was positively viewed by staff as this brought added value to the service and helped to ensure continuity and quality of patient care.

## Discussion

A key facet of Macmillan Specialist Care at Home is to achieve early referral into palliative care services to help staff initiate positive relationships and facilitate forward care planning with patients and their families [15]. In this study, early referrals were achieved for most people. There is, however, no room for complacency and our findings highlight that there is still work to be done in respect of earlier recognition of patient need to enable service provision before their conditions markedly deteriorate. Weakness and poor mobility were the most commonly reported symptoms here, and are corroborated elsewhere [40]. The rising symptom burden in the last year of life has been noted in recent research [41–43], and is compounded by co-morbidity and the changing demographics linked to an ageing population [44, 45]. Whilst existing research suggests that the trajectory of rising symptom burden is not unusual, our IPOS data indicated a slight decrease in symptom burden and, although not statistically significant (*p* = 0.3120), these findings point to high quality end of life care and support a trend towards achieving earlier intervention.

The evidence indicates that timely referral into palliative care services has a positive impact on quality of life and can reduce hospitalisation and symptom burden [46] but research in this area is largely North American [47, 48] or Japanese [49] and mainly relates to patients with cancer. Exceptions to this include investigative studies into symptom focused interventions for conditions such as breathlessness [50, 51]. The concept of ‘early referral’ is, however, imprecise, a factor that was reflected here in the different staff interpretations, both within, and across the six Innovation Centres. Further investigation may help to clarify this concept, particularly in relation to the UK health care context and for life-limiting conditions other than cancer.

In a similar vein, there is a lack of consensus both in the UK and internationally regarding the differences between specialist and generalist palliative care [52]. An important area for future attention is to agree core concepts as this will aid partnership working and support earlier access to the most appropriate forms of care [53–55]. Currently, the majority of people receive end of life from generalists such as GPs and district nurses rather than from those who have received specialist training in palliative care [53]. A key focus of recent UK policy is about improving palliative care provision through upskilling existing staff and increasing partnership working [2, 56]. Specialist services like Macmillan can play an important role in promoting the collaborative approach. As revealed in this study, it can be a sensitive arena for staff and requires careful management, training and education to allay fears about job security and promote an integrated approach whereby specialist and generalist workers can work harmoniously together to complement each other’s skills. Joint training is acknowledged as a crucial element in supporting the workforce and to increase palliative care knowledge amongst non-specialist services [57].

Figures from the evaluation’s VOICES-SF data on preferred place of death are comparative to existing national evidence [2] that indicate preferred place of death to be in one’s home, followed by inpatient hospices [58]. This position is reflected in UK health policy [59, 60]. However, it is important to note that numerous studies involving different health care systems and different patient groups have consistently shown that there is a discrepancy between expressed preferences for place of death and actual place of death [61, 62]. Recent evidence also challenges assumptions of home as the preferred place of death for all patients [5]. In this study, it was apparent that the input of Macmillan Specialist Care at Home helped patients to die in their preferred place of death, regardless of whether this was in the home, a hospice or other environment. This was shown to make a qualitative difference to patient experience and was appraised positively by bereaved family members. However, there is no room for complacency as a few responses revealed barriers to be overcome, particularly in respect of enabling home death. Significant here is to discover, and act on patient and family preferences as opposed to using place of death as a key indicator of quality of end of life care [5, 63]. Robinson et al. [64] emphasise that end of life decision making can be a complex affair and it can be particularly difficult for patients to exercise autonomy in the face of an uncertain and limited future [64]. A co-design approach to policy development in palliative care is proposed to increase the match between services and patient needs and wishes [65]. Macmillan cancer support care are key players in this respect, drawing on their expertise in public engagement to promote the lay voice.

The vast majority of community based palliative care is still related to symptom control, additional or enhanced support, coordinating care and end of life care [66, 67]. Moreover, there is little research that has formally evaluated or researched the impact of specialised interventions into home care. One exception is Morita et al. [68] assessing a comprehensive programme of interventions for specialist palliative care for people with cancer. This showed an increase in home deaths and patients and family members reported that quality of care increased. However, the interventions surveyed were education, specialist support and networking. None included specialised clinical interventions in the home environment. In this evaluation, the component of providing specialist interventions in the patient’s own home was not introduced across all sites and where it was, the time taken to set up and embed the provision was considerable. It is suggested that, in future, this aspect needs to be carefully weighted in relation to overall value and presents an area for further research to better clarify its potential.

Strong leadership to direct service provision and motivate staff working in challenging circumstances was a central finding in this research. Implementation leaders play a significant role in change management [69], and, in this study, project managers and consultants in palliative medicine were described as key agents. They provided a central focus for staff teams and proved essential to coordinate and manage the multiple and complex layers of intervention activity.

It was demonstrated here that, with proactive leadership, the challenges of managing a changing and expanding caseload can be addressed by using the team more flexibly and deploying staff differently. Macmillan Specialist Care at Home teams have a mandate to perform holistic care and see this as part of everyone’s role. Conferring a value on psychosocial aspects of care, and demonstrating it in multidisciplinary practice situations, can support and encourage flexibility in other professionals. This aspect was exemplified through the enhanced role of HCAs in Macmillan Specialist Care at Home and may reflect findings from other research [70] which suggests that their input is significant in community palliative care to complement existing provision.

Furthermore, incorporating a volunteer workforce was shown to add a valuable dimension to care. Volunteers can contribute across a range of areas including emotional, social, practical informal, and, if desired, spiritual/religious support [71]. These benefits impact on the organisation and the volunteers as well as the patients and carers concerned thus assessment of this component is suggested as a focal point for future investigation [72].

Realistic Evaluation offers a logical way of thinking about evaluation that allows for flexibility and creative use of methods to find the best evidence to answer the questions posed. It also acknowledges that a range of factors can affect care delivery and lead to variable outcomes between settings [73]. We encountered distinctive variations in the mode of implementation of Macmillan Specialist Care at Home across the six sites. This reflects the differential emphases given by each project team to the different components of the model but has also impacted on the level and quality of comparative findings that we could report in this evaluation. May et al. [74] discuss the need to understand complex interventions as non-linear, emergent and dynamic processes and the consequent implications for evaluation management and selection of feedback methods. Lessons learned here can be taken forward to shape further research and to ensure that greater coherence across study sites is achieved in future.

### Limitations

Each site was uniquely configured with no historical baseline of existing service provisions and their economic costs against which later comparison could be made. In addition, implementation of Macmillan Specialist Care at Home commenced at different times and evaluation data was subject to inconsistencies due to variable use and interpretation of the evaluation methods across the sites. Pressures on staff and turnover issues also affected data collection. This resulted in inconsistencies and absent data which raised specific challenges for data synthesis with the consequence that some measures were not considered reliable enough to report on. Whilst triangulation of the number of different data collections tools and data sources provided a much more robust and complete picture of the complexities of each site evaluated, care was required to ensure legitimate comparisons were made between the different data sets. A vast amount of data was generated overall, not all of which is reported on here but, using the Realist Evaluation [75] framework, this article draws together principal facets of the challenges and facilitators for project teams implementing Macmillan Specialist Care at Home in their area.

## Conclusions

Macmillan Specialist Care at Home is based on the four core components of early referral, home-based clinical interventions, close partnership working, and flexible teamwork. In this study early referral was achieved for the most part, leading to enhanced quality of end of life experience for many participants. The varied interpretations of terms including ‘early referral’ and ‘specialist’ and ‘generalist’ palliative care suggest a need for further clarification to help promote common understanding and to aid consistency in their future implementation. This is also key for evaluative work. The adoption of new, flexible modes of delivering palliative and end of life care present a range of challenges and sufficient time is needed to embed the necessary infrastructure. Important elements include joint education and training supported by strong project management and leadership. Findings show that, even in challenging circumstances, integrated, multidisciplinary teamwork can be achieved leading to positive benefits in respect of patient and carer needs in the community setting. Significant here were improved choice in respect of place of death and enhanced psycho-social support through health assistant rapid response and volunteering. The role of the consultants in palliative medicine helped to expedite clinical care and build staff confidence but specialist clinical interventions in the home, however, require further review to more fully assess the potential benefit to patients. Future research might focus on the provision of rapid response personnel to bridge gaps in services, such as out of hours, as these showed early indications of the potential to considerably improve patient and carer experience.

## Additional files

Additional file 1: Data collection methods for evaluating Macmillan Specialist Care at Home (DOC 34 kb)

Additional file 2: Breakdown of data collection by Innovation. (DOCX 13 kb)

Additional file 3: Demographic and clinical details of patients referred to Macmillan Specialist Care at Home. (DOCX 13 kb)

Additional file 4: Referral to the six Macmillan Specialist Care at Home services – SDT data^a^. (DOCX 13 kb)

Additional file 5: Survival time as indicated by PPI score at referral. (DOCX 15 kb)

Additional file 6: Patient reported symptom burden - IPOS data. (DOCX 78 kb)

Additional file 7: Place of death by site - SDT data. (DOCX 27 kb)

Additional file 8: Carers’ perceptions of need - CSNAT tool. (DOCX 16 kb)

Additional file 9: Meeting the patient need – VOICES-SF questionnaire. (DOCX 156 kb)

## Abbreviations

CSNAT: Carers Support Needs Assessment Tool
HCA: Health Care Assistant
IPOS: Integrated Palliative Outcome Scales
KPS: Karnofsky Performance Scale
PPI: Palliative Prognostic Index
PPS: Palliative Performance Scale
SDT: Service Data Tool
VOICES-SF: Views of Informal Carers – Evaluation of Services: Short Form

## Footnotes

1 The health care assistants (HCAs) provided a range of supportive duties. These included observing, monitoring and recording patients’ conditions by taking vital measures such as pulse, temperature and respirations, and assisting with clinical interventions such as venepuncture. Other important role dimensions involved communication with patients, relatives and carers, providing information and undertaking personal care with patients.
