# Supplementary material for: Impact of the Macmillan specialist Care at Home service: a mixed methods evaluation across six sites
Source: BMC Palliat Care. 2018 Feb 23;17:36. doi: 10.1186/s12904-018-0281-9 (PMC6389143; doi:10.1186/s12904-018-0281-9)
Supplement: Supplementary file 1 — Data collection methods for evaluating Macmillan Specialist Care at Home (DOC 34 kb) [file 12904_2018_281_MOESM1_ESM.doc]

**Additional File 1** Data collection methods for evaluating Macmillan Specialist Care at Home

| **Contextual Factors**  Macmillan Cancer Support objectives and national stakeholder interview messages  Six Innovation Sites –demographics, socio-economic status, geography, existing services  Macmillan Specialist Care at Home: 1) Service characteristics; 2) Outer settings; 2) Inner setting; 3) Characteristics of individuals involved; 4) Process of implementation | | | |
| --- | --- | --- | --- |
| Qualitative data | Site visits (n=6)  Stakeholder interviews (n=5)  Project team and staff focus groups (n=9) participants (n=62) | Site visits – project mapping  Project team and staff focus groups (n=11) participants (n=70)– service developments and work patterns/changes  Volunteer focus groups (n=3) participants (n=14) – perception and experience of role  Local stakeholder interviews (n=6)  *Pictor^1, 2^ interviews (n=49) with patients (n=9), carers (n=8), volunteers and staff (n=32) to explore collaborative working practices and roles and identities | Site visits  Project team and staff focus groups (n=12)  participants (n=58)  Volunteer focus groups (n=3)  participants (n=18) |
| Quantitative testing of patient, carer and staff experience. |  | SDT (n=88) – referrals, place of death, staff activity logs, clinical interventions and their location  PPS^3^, PPI^4, 5^ (n=2,711 [response rate 88.8%]) – patient symptom burden, physical function, survival time  IPOS^6^ (n=1,157 [response rate 37.9%])– patient physical symptoms, psychological, emotional, spiritual, information and support needs  CSNAT^7^ – carers (n=241)  VOICES-SF^8, 9^ – bereaved carers (n=102 [response rate 34.7%]) | Monitoring data |
| Hypothesis | Baseline  Macmillan Specialist Care at Home leads to enhanced quality of end of life care provision | Interim  Service Implementation  Additional staffing/volunteer recruitment  Education and training  Service integration/new models of working | Final |

Mechanisms of impact

Participant/Carer/Staff responses to and interactions with the intervention

Mediators/Champions

Unexpected pathways and consequences

Outcomes

1. Burr V, King N and Butt T. Personal construct psychology methods for qualitative research. *International Journal of Social Research Methodology*. 2014; 17: 341-55.

2. King N, Bravington A, Brooks J, Hardy B, Melvin J and Wilde D. The Pictor technique: a method for exploring the experience of collaborative working. *Qual Health Res*. 2013; 23: 1138-52.

3. Ho F, Lau F, Downing M and Lesperance M. A reliability and validity study of the Palliative Performance Scale. *BMC Palliative Care*. 2008; 7: 10.

4. Morita T, Tsunoda J, Inoue S and Chihara S. Improved accuracy of physicians' survival prediction for terminally ill cancer patients using the Palliative Prognostic Index. *Palliat Med*. 2001; 15: 419-24.

5. Stone CA, Tiernan E and Dooley BA. Prospective validation of the palliative prognostic index in patients with cancer. *J Pain Symptom Manage*. 2008; 35: 617-22.

6. Cicely Saunders Institute. Integrated Palliative Outcome Scale (IPOS) in English. *Palliative Care Outcome Scale: a resource for palliative care*. Cicely Saunders Institute, 2012.

7. Ewing G, Austin L, Diffin J and Grande G. Developing a person-centred approach to carer assessment and support. *British Journal of Community Nursing*. 2015; 20: 580-4.

8. Hunt KJ and Addington-Hall J. A toolkit for the design and planning of locally-led VOICES end of life care surveys. Southhampton: University of Southampton, 2011.

9. Burt J, Shipman C, Richardson A, Ream E and Addington-Hall J. The experiences of older adults in the community dying from cancer and non-cancer causes: a national survey of bereaved relatives. *Age Ageing*. 2010; 39: 86-91.
