# Supplementary material for: Impact of the Macmillan specialist Care at Home service: a mixed methods evaluation across six sites
Source: BMC Palliat Care. 2018 Feb 23;17:36. doi: 10.1186/s12904-018-0281-9 (PMC6389143; doi:10.1186/s12904-018-0281-9)
Supplement: Supplementary file 2 — Breakdown of data collection by Innovation. (DOCX 13 kb) [file 12904_2018_281_MOESM2_ESM.docx]

**Additional File 2** Breakdown of data collection by Innovation Centre

| **Data collection tools** | **Sites** | | | | | | |
| --- | --- | --- | --- | --- | --- | --- | --- |
|  | **Site A** | **Site B** | **Site C** | **Site D** | **Site E** | **Site F** | **Total** |
| **Qualitative data collection** |  | | | | | | |
| ***Project team and staff focus groups***  **(n= total number of participants)** | 4 groups  (n=22) | 7 groups  (n=48) | 6 groups  (n=33) | 5 groups  (n=24) | 6 groups  (n=26) | 5 groups  (n=37) | **33 groups**  **(n=190^a^)** |
| ***Local stakeholder interviews*** | 1 | 1 | 0 | 2 | 1 | 1 | **6** |
| ***Volunteers focus groups***  **(n= total number of participants)** | 1 group  (n=10) | 1 group  (n=4) | 1 group  (n=4) | 1 group  (n=4) | 1 group  (n=6) | 1 group  (n=4) | **6 groups**  **(n=32)** |
| ***Pictor*** | 9 | 8 | 8 | 9 | 7 | 8 | **49** |
| ***National Stakeholder Interviews*** | n/a | | | | | | **5** |
| **Quantitative outcome measures^b^** |  | | | | | | |
| ***SDT*** (Completed monthly) | 15 (100%) | 13 (86.7%) | 15 (100%) | 15 (100%) | 15 (100%) | 15 (100%) | **88 (97.8%)** |
| ***PPI & PPS*** | 0 | 1,612 (84.5%) | 92 (78.0%) | 589 (100%) | 228 (100%) | 190 (86.4%) | **2,711 (88.8%)** |
| ***IPOS*^a^** | 0 | 692 (36.3%) | 82 (69.5%) | 177 (31.2%) | 124 (55.1%) | 82 (37.1%) | **1,157 (37.9%)** |
| ***VOICES*** | 0 | 0 | 2 (4.6%) | 62 (50.4%) | 16 (22.2%) | 22 (39.3%) | **102 (34.7%)** |
| ***CSNAT*^c^** | 0 | 180 | 30 | 9 | 2 | 20 | **241** |

^a^This is total number of participants, as staff took part in more than one focus group this figure does not represent total number of individuals participating

^b^The denominator for the SDT response rate is 15, the maximum number of months that sites were asked to complete the tool. The denominator for PPI, PPS and IPOS is the number of patients who were accepted by the service and received care. The VOICES questionnaire was sent to a sample of bereaved carers and sites kept a record of who was sent a questionnaire and this was used as the denominator.

^c^Response rates are not shown for CSNAT as the sites were unable to report how many carers were invited to complete the questionnaire.
