# Supplementary material for: Impact of the Macmillan specialist Care at Home service: a mixed methods evaluation across six sites
Source: BMC Palliat Care. 2018 Feb 23;17:36. doi: 10.1186/s12904-018-0281-9 (PMC6389143; doi:10.1186/s12904-018-0281-9)
Supplement: Supplementary file 3 — Demographic and clinical details of patients referred to Macmillan Specialist Care at Home. (DOCX 13 kb) [file 12904_2018_281_MOESM3_ESM.docx]

**Additional File 3** Demographic and clinical details of patients referred to Macmillan Specialist Care at Home - PPI&PPS data

|  | **Site B** | **Site C** | **Site D** | **Site E** | **Site F** | **All sites** |
| --- | --- | --- | --- | --- | --- | --- |
| **Mean Age (SD)** | 76.5  (12.5) | 68.6  (15.2) | 75.5  (11.7) | 72.9  (13.4) | 75.9  (10.2) | **75.7**  **(12.5)** |
| **Gender (%)** | | | | | | |
| Women | 996  (49.8) | 50  (49.5) | 280  (43.3) | 161  (56.9) | 87  (44.4) | **1,574 (48.7)** |
| Men | 1,006  (50.2) | 51  (50.5) | 367  (56.7) | 122  (43.1) | 109  (55.6) | **1,655 (51.3)** |
| **Diagnosis (%)** | | | | | | |
| Cancer | 1,133  (57.2) | 82  (81.2) | 539  (84.0) | 241  (86.1) | 166  (84.7) | **2,161**  **(67.6)** |
| Non-Cancer | 847  (42.8) | 19  (18.8) | 103  (16.1) | 39  (13.9) | 30  (15.3) | **1,038**  **(32.5)** |
| Neurological Conditions | 162  (8.2) | 11  (10.9) | 17  (2.8) | 9  (3.3) | 3  (1.6) | **202**  **(6.4)** |
| Dementia / Frailty | 173  (8.7) | 3  (3.0) | 19  (3.1) | 5  (1.8) | 1  (0.5) | **201**  **(6.4)** |
| Cardiac Conditions | 146  (7.4) | 2  (2.0) | 11  (1.8) | 7  (2.6) | 7  (3.7) | **173**  **(5.5)** |
| Respiratory Conditions | 129  (6.5) | 2  (2.0) | 13  (2.1) | 8  (2.9) | 10  (5.3) | **162**  **(5.1)** |
| Multiple Co-morbidities | 30  (1.5) | 0  (0.0) | 1  (0.2) | 0  (0.0) | 0  (0.0) | **31**  **(1.0)** |
| Other | 206  (10.4) | 1  (1.0) | 10  (1.6) | 2  (0.7) | 2  (1.1) | **221**  **(7)** |
