# Supplementary material for: Impact of the Macmillan specialist Care at Home service: a mixed methods evaluation across six sites
Source: BMC Palliat Care. 2018 Feb 23;17:36. doi: 10.1186/s12904-018-0281-9 (PMC6389143; doi:10.1186/s12904-018-0281-9)
Supplement: Supplementary file 4 — Referral to the six Macmillan Specialist Care at Home services – SDT dataa. (DOCX 13 kb) [file 12904_2018_281_MOESM4_ESM.docx]

Additional File 4 Referral to the six Macmillan Specialist Care at Home services – SDT data^a^

|  | **Site A** | **Site B** | **Site C** | **Site D** | **Site E** | **Site F** | **All sites** |
| --- | --- | --- | --- | --- | --- | --- | --- |
| Number of referrals received | 15 | 1,998 | 135 | 639 | 226 | 273 | **3,286** |
| Number of referrals not accepted by the service (%) | 0 (0.0) | 5 (0.3) | 12 (8.9) | 14 (2.1) | 1 (0.4) | 14 (5.1) | **46 (1.4)** |
| Number of patients who died before any care provided (%) | 0 (0.0) | 86 (4.3) | 5 (3.7) | 16 (2.5) | 0 (0.0) | 38 (13.9) | **145 (4.4)** |
| Number of patients who received care from a Macmillan Specialist Care at Home service (%) | 15 (100) | 1,907 (95.4) | 118 (87.4) | 625 (97.8) | 225 (99.6) | 221 (81.0) | **3,095 (94.2)** |

^a^Data generated from the 15 months between the 01/11/14 and the 31/01/16 as reported by sites in the SDT
