# Supplementary figures and images for: Impact of the Macmillan specialist Care at Home service: a mixed methods evaluation across six sites
Source: BMC Palliat Care. 2018 Feb 23;17:36. doi: 10.1186/s12904-018-0281-9 (PMC6389143; doi:10.1186/s12904-018-0281-9)

**Additional File 6**


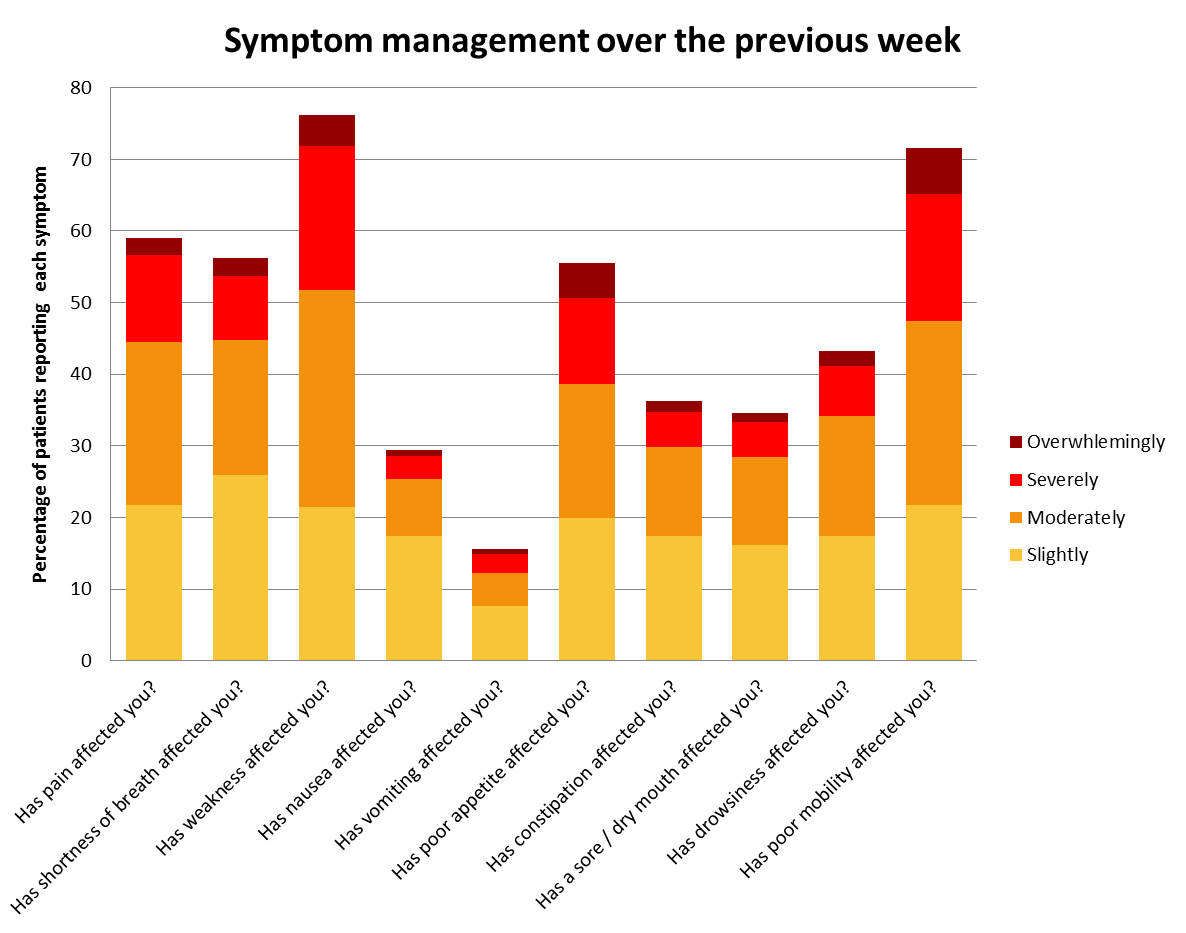

Supplement: Supplementary file 6 — Patient reported symptom burden - IPOS data. (DOCX 78 kb) [file 12904_2018_281_MOESM6_ESM.docx]

**Additional File 9**


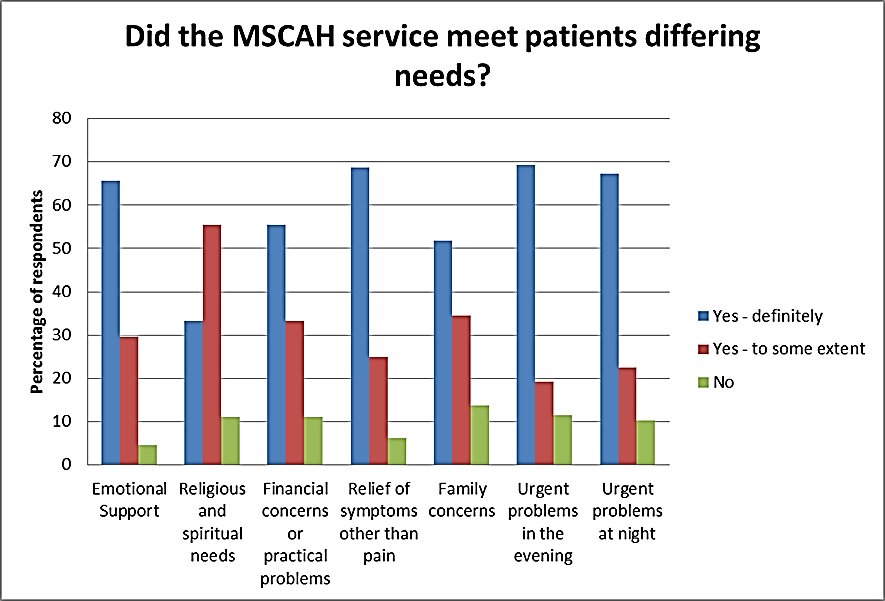

Supplement: Supplementary file 9 — Meeting the patient need – VOICES-SF questionnaire. (DOCX 156 kb) [file 12904_2018_281_MOESM9_ESM.docx]
