# Supplementary material for: Impact of the Macmillan specialist Care at Home service: a mixed methods evaluation across six sites
Source: BMC Palliat Care. 2018 Feb 23;17:36. doi: 10.1186/s12904-018-0281-9 (PMC6389143; doi:10.1186/s12904-018-0281-9)
Supplement: Supplementary file 8 — Carers’ perceptions of need - CSNAT tool. (DOCX 16 kb) [file 12904_2018_281_MOESM8_ESM.docx]

**Additional File 8**
